# A Novel Approach to Realize Plasmonic Sensors via Multimode Optical Waveguides: A Review

**DOI:** 10.3390/s23125662

**Published:** 2023-06-17

**Authors:** Francesco Arcadio, Domenico Del Prete, Luigi Zeni, Nunzio Cennamo

**Affiliations:** Department of Engineering, University of Campania Luigi Vanvitelli, Via Roma 29, 81031 Aversa, Italy; domenico.delprete@unicampania.it (D.D.P.); luigi.zeni@unicampania.it (L.Z.); nunzio.cennamo@unicampania.it (N.C.)

**Keywords:** surface plasmon resonance (SPR), multimode waveguides, multimode optical fibers, plastic optical fibers (POFs), optical fiber sensors

## Abstract

In recent decades, the Surface Plasmon Resonance (SPR) phenomenon has been utilized as an underlying technique in a broad range of application fields. Herein, a new measuring strategy which harnesses the SPR technique in a way that is different from the classical methodology was explored by taking advantage of the characteristics of multimode waveguides, such as plastic optical fibers (POFs) or hetero-core fibers. The sensor systems based on this innovative sensing approach were designed, fabricated, and investigated to assess their ability to measure various physical features, such as magnetic field, temperature, force, and volume, and to realize chemical sensors. In more detail, a sensitive patch of fiber was used in series with a multimodal waveguide where the SPR took place, to alter the mode profile of the light at the input of the waveguide itself. In fact, when the changes of the physical feature of interest acted on the sensitive patch, a variation of the incident angles of the light launched in the multimodal waveguide occurred, and, as a consequence, a shift in resonance wavelength took place. The proposed approach permitted the separation of the measurand interaction zone and the SPR zone. This meant that the SPR zone could be realized only with a buffer layer and a metallic film, thus optimizing the total thickness of the layers for the best sensitivity, regardless of the measurand type. The proposed review aims to summarize the capabilities of this innovative sensing approach to realize several types of sensors for different application fields, showing the high performances obtained by exploiting a simple production process and an easy experimental setup.

## 1. Introduction

Optical sensors have been developed recently using several sensing principles, such as photonic crystals, which are an attractive sensing approach for realizing optical sensors and biosensors [1,2,3]. However, these types of sensors require expensive technologies to develop them.

In recent years, the Surface Plasmon Resonance phenomenon has achieved a lot of importance in different application fields. This is a highly sensitive technique that allows the measurement of a small refractive index variation at the boundary between a metallic layer and an encompassing dielectric medium. Once the resonance conditions are verified, a dip in the output signal, as a consequence of a strong absorption, can be observed. The dip is achieved at a particular wavelength, called the resonance wavelength, and it is linked to the refractive index at the boundary [4,5,6,7,8]. This technique can be paired with optical waveguides, such as optical fibers, to attain sundry sensor systems for several application areas [9,10,11,12,13]. These types of sensors have important advantages, such as low cost, rapid response, immunity to electromagnetic interference, high sensitivity, and small size. 

In particular, the SPR sensor can be used as a refractometer, a bio/chemical sensor by functionalizing the surface with a particular receptor, a pressure sensor, and so on [14,15,16,17,18,19,20]. Generally, the sensor system based on the SPR can be realized by using different types of optical waveguides, such as monomodal or multimodal. Thanks to the properties of the multimodal waveguides, the SPR technique can be exploited more efficiently, and, for this reason, multimodal waveguides are widely adopted for optical sensors [12]. In more detail, when dealing with multimodal waveguides, the dip, caused by the SPR phenomenon, is wider when compared with that caused by the monomodal waveguides. This phenomenon is a consequence of a convolution of diverse propagating modes, each of which is linked to a distinct angle of incidence at the metal–dielectric interface [12,21]. In such a case, despite broader SPR spectra being obtained, a greater sensitivity is achieved as well, thus leading to an overall performance improvement [12].

In this review, a focus on a new sensing method based on the properties of the multimode waveguides to excite SPR phenomena is reported. The overview aims to summarize the capabilities of this innovative sensing approach to realize several types of sensors for different application fields. The multimode waveguides allow the tuning of the resonance wavelength by varying the mode profile of the input light and, consequently, the incident angles of the modes propagating into an SPR chip. In this way, innovative optical fiber sensors can be realized, showing high performances by exploiting simple production processes and an easy experimental setup. More specifically, several sensor systems based on this novel sensing approach are presented in this review. In general, the sensor system consists of a first section sensitive to the alterations of the measurand of interest (such as a magnetic field, temperature, force, or analyte). The second section is represented by an SPR D-shaped POF platform, extensively presented in the literature [22], or a different SPR platform based on multimodal waveguides (such as POFs, hetero-core fibers, slab waveguides, or specialty optical fibers) to trigger the SPR phenomena. Hence, these two components (sensing chip and SPR chip) forming the sensor system are connected in a series between a white light source and a spectrometer. The sensing principle is the same for all the sensors based on this approach: the refractive index of the solution on the SPR chip is unchanged, whereas the launching condition of the light inputting the plasmonic chip is modulated by the quantity of interest interacting with a sensitive patch (first chip). In this way, it is possible to optimize the total thickness of the layers deposited on the plasmonic platform (regardless of the sensitive layer, which is no longer present in this section) toward the best resonance condition. 

Concerning the experimental configurations reported in this review, different tests were carried out in order to characterize the sensor systems in terms of sensitivity and resolution. The proposed approach was tested for physical quantities (e.g., magnetic field, temperature, force, volume, and displacement) and for chemical sensing applications as well. Furthermore, an analysis was performed for each presented sensor to compare its performance with state-of-the-art experimental configurations.

## 2. Background on the Sensing Principle and Experimental Setup

The proposed sensing approach harnesses the properties of multimodal waveguides to tune opportunely the SPR phenomenon. As previously stated, the sensor systems based on this method consist of two main parts: the first is a transducer realized by using a patch purposely modified to make it sensitive to a specific quantity; the second consists of a plasmonic platform based on multimodal waveguides, which is connected in series to the sensitive patch. 

The sensor systems are tested by varying the measurand, which interacts with the sensitive patch, and by fixing the refractive index of the dielectric medium on the SPR platform. In fact, once a variation of the measurand takes place, a change in the sensitive patch occurs, thus leading to an alteration in the propagating modes in the plasmonic platform. This variation, related to the change of the quantity under test, varies the angles of incidence connected with the propagating modes in the plasmonic platform. In this way, the resonance condition varies; hence, the resonance wavelength shifts.

The experimental equipment adopted to investigate the mentioned sensor systems consists of a broadband light source and a spectrometer, whose working range can be in the visible range, such as in the case of SPR sensors based on plastic optical fibers (POFs), or in the telecom window, such as in the case of silica-fiber-based plasmonic sensors. The series between the sensitive patch and the SPR platform is located between the light source and the spectrometer. In particular, the light source is connected to the measurand-sensitive patch; the latter is then coupled to the plasmonic platform, whereas the spectrometer collects the light exiting the SPR sensor. In the end, a laptop, connected to the spectrometer, is used to attain and process the data.

**Figure 1:**
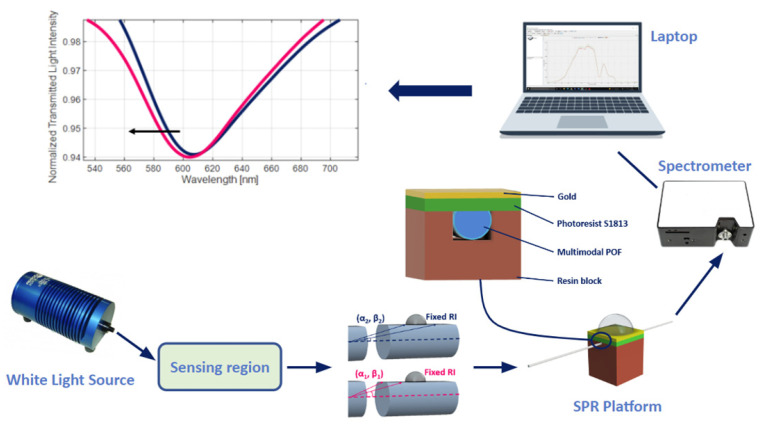
Outline of a sensor system based on an SPR-POF platform, which exploits the novel sensing approach. Once a variation of the mode profile occurs following the interaction between the sensitive patch and the quantity under test, the SPR wavelength shifts (for instance, from blue to magenta curve).

As an example, Figure 1 reports a schematization of the sensor system based on an SPR-POF sensor [22]. In particular, the SPR D-shaped platform is realized by using a POF with a core diameter of 980 μm and a cladding of 10 μm (total diameter of about 1 mm). The POF is glued inside a resin block, used as a support, and polished by using two types of lapping sheets (5 and 1 μm grits) to obtain a D-shaped area. After this, a buffer layer (Microposit S1813, Allresist GmbH, Strausberg, Germany) is deposited on the modified POF by spin coating, and, finally, a thin gold layer (60 nm of thickness) is deposited on the buffer layer by means of a sputtering process [22].

The experimental setup used to test this configuration comprises a white light source and a spectrometer. In particular, the white light source (HL–2000–LL, Ocean Optics, Dunedin, FL, USA) has a wavelength emission range from 360 nm to 1700 nm, and the spectrometer (FLAME-S-VIS-NIR-ES, Ocean Optics, Dunedin, FL, USA) has a detection range between 350 nm and 1000 nm. 

In this review, several sensor systems based on this sensing approach are recalled. In particular, physical and bio/chemical sensors are reported via two specific sections. 

## 3. Physical Sensing

### 3.1. Magnetic Field Sensor

**Figure 2:**
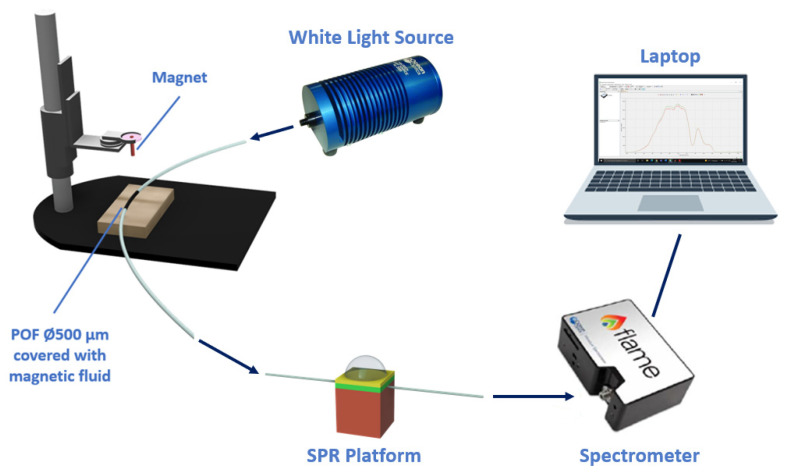
Outline of the magnetic field sensor system. A special holder is utilized to tune the distance between the magnet and the ferrofluidic-covered patch.

The magnetic field sensor is realized by using a POF patch, with a core diameter of 500 μm, paired with the SPR-POF sensor. To make the patch sensitive to the change in the magnetic field, it is coated with a ferrofluid along 20 mm of its length [23]. To modify the mode profile of the input light, a magnet is used, since, by tuning the distance from the sensitive patch, it is possible to change the value of the bending force on the patch covered with ferrofluid. The distance between the magnet and the sensitive patch can be regulated by a special holder, as schematically reported in Figure 2. 

**Figure 3:**
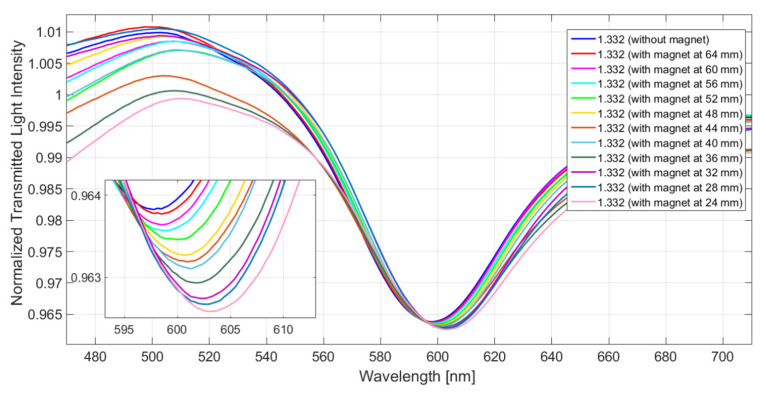
Normalized transmitted spectra obtained with water (*n* = 1.332) on the plasmonic platform and by varying the spacing between the magnet and the ferrofluidic-covered patch. Reprinted with permission from Ref. [23]. Copyright 2020, IEEE.

The sensor system is tested by keeping unchanged the refractive index on the plasmonic platform (equal to 1.332, i.e., water) and by tuning the distance between the magnet and the ferrofluidic-covered patch, thus making it possible to change the intensity of the target magnetic field. More details about this magnetic field sensor are extensively reported in [23]. The analyzed magnetic field ranges from 0.15 mT to 1.20 mT, which corresponds, in terms of spacing between the magnet and the sensitive patch, to a range between 64 mm and 24 mm, with a step size of 4 mm. Therefore, once the magnet approaches the ferrofluidic-covered area, the intensity of the exited magnetic field increases. Figure 3 shows the spectra obtained by normalizing the transmission spectra on the reference, acquired with water (*n* = 1.332) on the SPR platform and without the magnet around the POF covered with ferrofluid. The results show that the resonance wavelength increases with the magnetic field values.

**Figure 4:**
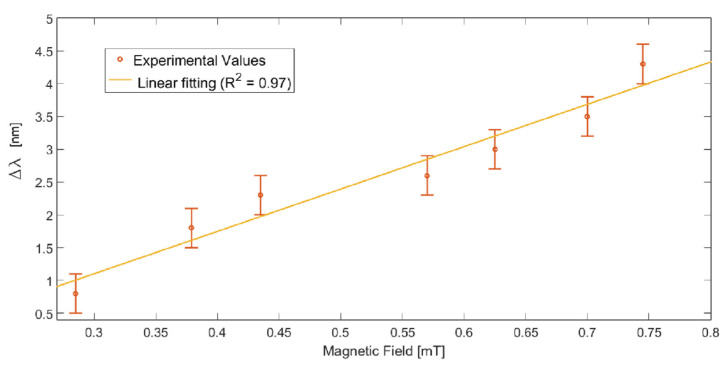
Experimental variation in resonance wavelength, calculated with respect to the condition without magnet around the ferrofluidic-covered patch, in the range from 0.15 mT to 0.7 mT. The linear fitting of the experimental values and the error bars are also reported. Reprinted with permission from Ref. [23]. Copyright 2020, IEEE.

More specifically, the magnetic field sensor system denotes a good linear response in the magnetic field intensity range between 0.28 mT and 0.75 mT, as reported in Figure 4. The latter reports the experimental variations in resonance wavelength calculated with respect to the resonance wavelength related to the reference (acquired without the magnet) in the analyzed magnetic field range. In this range, the sensitivity can be calculated as reported in [23] by using the linear fitting reported in Figure 4, resulting in approximately 6800 pm/mT, with a resolution equal to approximately 0.029 mT [23].

An analysis, reported in Table 1, is carried out to compare the presented magnetic field platform, with other configurations already presented in the literature.

### 3.2. Temperature Sensor

The temperature sensor system includes two D-shaped-POF probes which are coupled in a sequence: the first platform is characterized by a coating of thermosensitive material on the exposed POF core to make it responsive to temperature changes, while the second one consists of an SPR-POF probe [27].

The POF used for the realization of both the platforms is multimodal, with a 1 mm diameter. The production process of the thermosensitive platform can be summarized as follows. First, the POF is fixed with glue onto a physical support, and then it is polished with two types of lapping sheets (5 and 1 μm grits) in order to obtain the D-shaped area. On the modified POF, a layer of thermosensitive material is then deposited. For this purpose, silicone is used since its physical characteristics are dependent on the temperature [28]. 

As a proof of concept to realize the thermal POF sensor chip, an adhesive tape with a nominal thickness of 70 μm is used to control the thickness of the silicone layer. More specifically, by exploiting the adhesive tape, a channel is achieved by fixing two pieces of adhesive tape at the end of the physical support. After this, the silicone is placed on the exposed POF’s core by exploiting a spatula to level the silicone layer at the adhesive tape thickness. In this way, a silicone thickness of 70 μm is obtained. This simple approach offers the ability to realize thermal sensor chips with good reproducibility. 

The sensor’s performances can be changed by using a different thickness of the silicone layer. 

**Figure 5:**
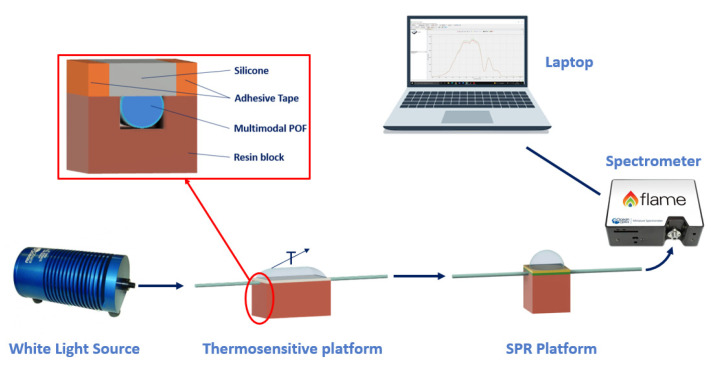
Outline of the temperature (T) sensor system. Inset: details of the thermosensitive probe.

Figure 5 shows a schematization of the temperature sensor system with a focus on the cross section of the thermosensitive platform.

**Figure 6:**
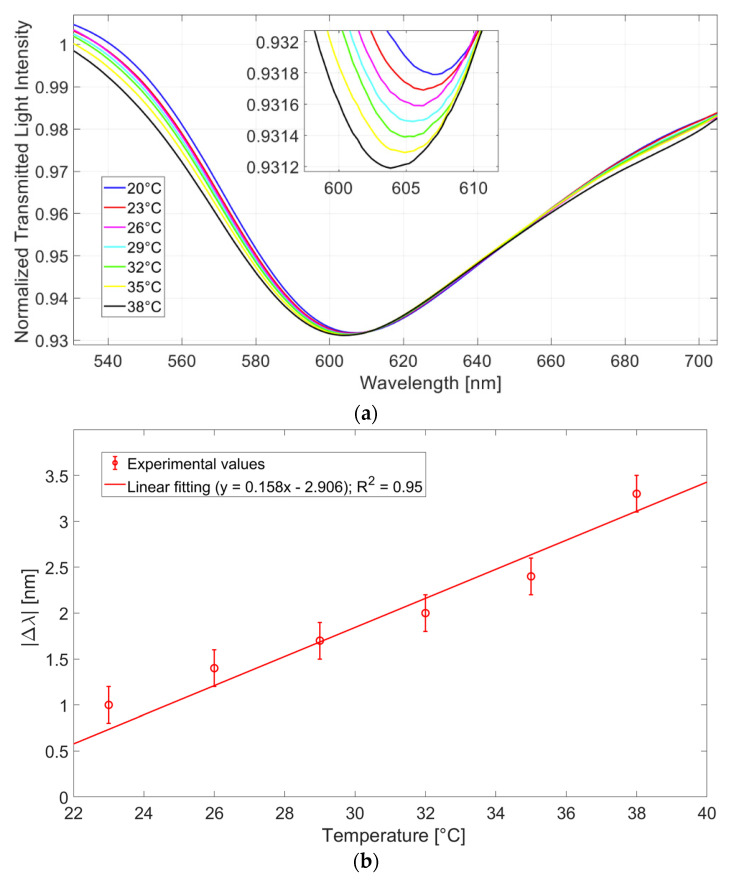
(**a**) SPR spectra obtained by keeping unchanged the refractive index of the solution on the plasmonic probe to 1.332 and by varying the temperature of the water upon the thermosensitive platform from 20 °C to 38 °C, with a step size of 3 °C [27]. (**b**) Experimental wavelength variations, in absolute value, computed with respect to the value obtained with water at 20 °C, as a function of the temperature of the water upon the thermosensitive platform. The linear fitting and the error bars are also reported [27]. Reprinted with permission from Ref. [27]. Copyright 2022, IEEE.

The presented sensor system is tested by tuning the temperature of the water deposited on the thermosensitive platform to induce a change in the physical characteristics and, consequently, in the refractive index, of the silicone layer [28]. During the test, the refractive index of the solution upon the SPR D-shaped platform is fixed at 1.332 (water), and the temperature of the solution upon the thermosensitive platform varies from 20 °C to 38 °C, with a step size of 3 °C. More details about this temperature sensor are extensively reported in [27]. Figure 6a shows the normalized SPR spectra obtained by tuning the temperature of the water on the thermosensitive platform, whereas Figure 6b reports the absolute values of the resonance wavelength variation, calculated with respect to the value obtained with water at 20 °C versus the temperature values of the water upon the thermosensitive platform. In Figure 6b, the linear fitting of the experimental data and the error bars are also reported. From the results in Figure 6, it is worth noting that once the temperature of the solution increases, the resonance wavelength decreases (blue shift). In more detail, the sensor denotes an acceptable linear behavior, and the sensitivity, estimated by using the linear fitting equation reported in Figure 6b, is approximately 0.158 nm/°C, while the resolution is 1.2 °C [27].

An analysis, reported in Table 2, is performed to compare the presented temperature sensor platform with other configurations already presented in the literature.

### 3.3. Force Sensor

**Figure 7:**
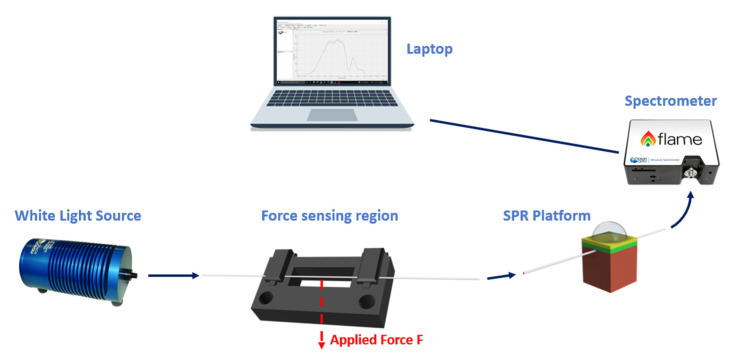
A schematization of the force sensor system.

The force sensor system is realized by using a 1 mm POF patch immobilized into a 3D-printed support through two clamps and representing the force sensing region. The force sensitive patch is then coupled to an SPR-POF probe in a series. The holder, reported in Figure 7, is devised and successively produced by means of a 3D printer (Photon Mono X, Anycubic^®^, Shenzhen, China) [32].

The POF patch is subject to various forces by placing various weights in the center of the sensing area. In the same way as the previous sensor systems, the refractive index of the solution on the SPR platform is fixed. By varying the applied force, the incidence angles related to the propagating modes in the following SPR-POF platform change, and so the resonance wavelength is tuned. Figure 7 reports a schematization of the abovementioned force sensor system.

**Figure 8:**
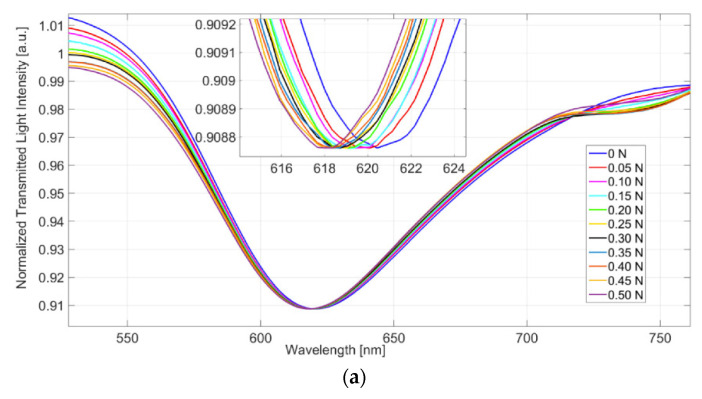

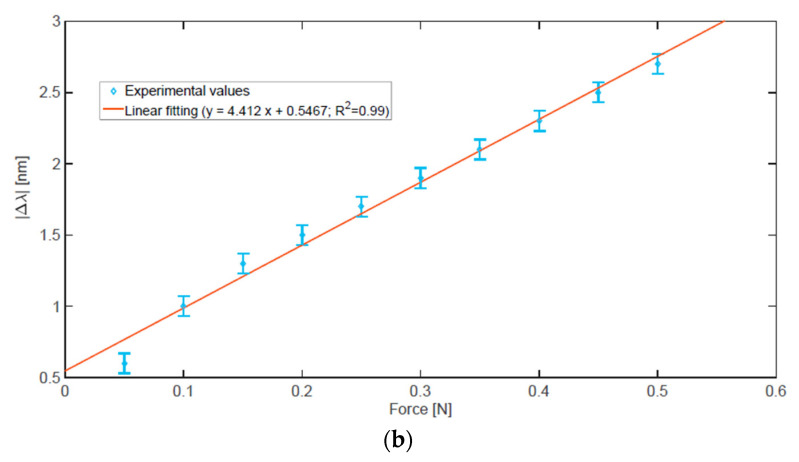
(**a**) Normalized transmitted spectra obtained by a normalization of the spectrum achieved with no applied force in the force sensing region and with air on the SPR platform. The analyzed range is between 0 and 0.5 N, with a step size of 0.05 N. (**b**) Experimental wavelength variation, in absolute value, versus the applied force in the range from 0 to 0.5 N, with a step size of 0.05 N. Linear fitting of the experimental data and error bars are reported as well [32].

The force sensor system is tested by varying the applied force to the POF patch in the range between 0 N and 0.5 N (with a step size of 0.05 N) and by fixing the refractive index of the solution on the SPR platform to 1.332 (water). More details about this force sensor are extensively reported in [32]. Figure 8a shows the transmitted spectra obtained by normalization of the spectrum attained without any applied force and with air on the SPR platform, in the range previously mentioned. From Figure 8a, a clear blue-shift can be observed, since the resonance wavelength decreases with the increasing of the applied force. In the considered force range (0–0.5 N), the devised sensor indicates a good linear behavior, denoting a sensitivity and a resolution of 4.4 nm/N and 22 mN, respectively [32]. These values can be estimated by using the linear fitting of the experimental wavelength variation, reported in Figure 8b, calculated with respect to the resonance wavelength acquired without the applied force. Furthermore, in Figure 8b, the error bars of the experimental values are also reported by considering the maximum measured standard deviation (0.1 nm) [32]. 

A comparative analysis in terms of resolution, reported in Table 3, is carried out by comparing different types of force sensors already presented in the literature. 

### 3.4. Micro-Liquid Volume Sensor

The micro-liquid volume sensor system consists of a patch of light diffusive fiber (LDF). In LDFs, the light is spread from the core, and it is diffused toward the external medium thanks to the scattering centers present in their core. The used LDF-POF is fixed in a 3D-printed tank in order to realize the volume-sensing region, and it is coupled in a sequence with an SPR-POF probe [36].

**Figure 9:**
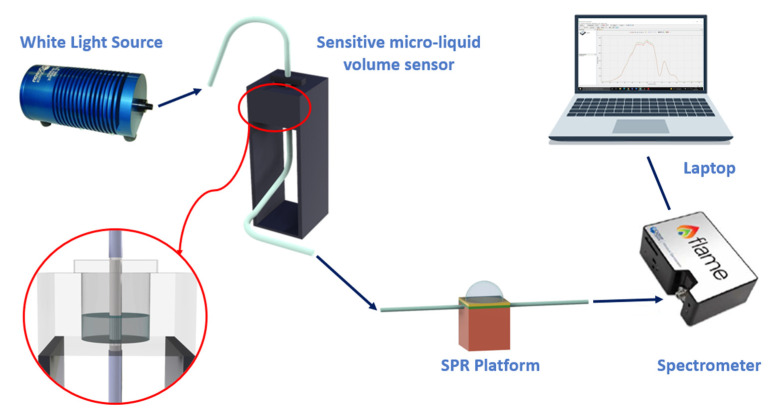
Outline of the micro-volume liquid sensor system.

The tank, highlighted in the outline of the micro-liquid sensor system in Figure 9, is printed using a 3D printer (Photon Mono X, Anycubic, Shenzhen, China), and its aim is to hold the micro-liquid volume around the fiber. The patch consists of a 1600 μm uncladded LDF-POF (manufactured by Global Engineering Network, Dosson di Casier, Italy) with a removable jacket of approximately 400 μm (total diameter 2 mm). The jacket is stripped off by a mechanical tool along 1 cm of its length; this piece of fiber without jackets represents the volume-sensitive area, and it is fixed in the tank through two holes using white silicone [36]. 

The mode profile of the input light at the SPR platform is altered by changing the surrounding volume of liquid in the tank. In this way the resonance wavelength changes, and it is possible to detect small variations of volume [36]. More details about this micro-liquid volume sensor are extensively reported in [36].

**Figure 10:**
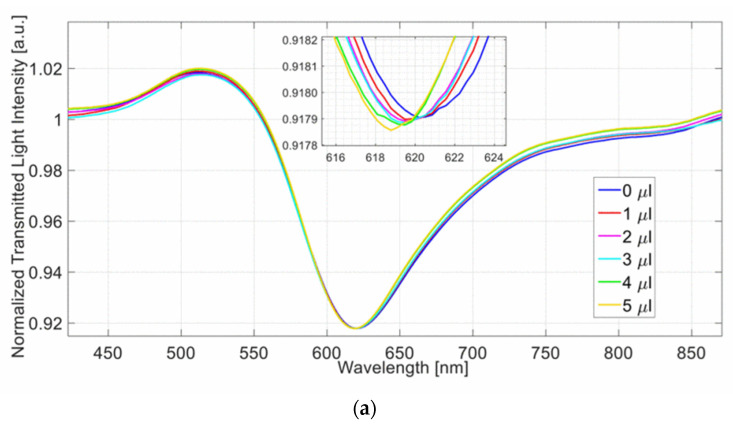

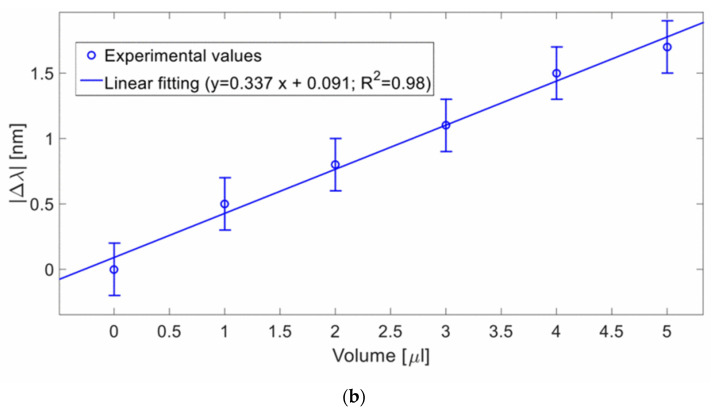
(**a**) SPR spectra achieved by fixing the refractive index on the SPR platform to 1.332 (water) and by changing the liquid volume in the tank from 0 to 5 μL. (**b**) Absolute values of the resonance wavelength variation, computed with respect to the condition without water volume surrounding the modified LDF-POF, as a function of the water volume that surrounded the LDF patch. Linear fitting of the experimental data and error bars are reported as well. Reprinted with permission from Ref. [36]. Copyright 2022, IEEE.

The presented sensor system is tested by fixing the refractive index of the solution on the SPR platform to 1.332 (water) and by changing the water volume in the tank, ranging from 0 to 5 μL (1 μL step). Figure 10a shows the transmitted spectra obtained by a normalization of the spectrum acquired with the LDF patch dry and air as the surrounding medium of the SPR-POF platform. Figure 10b reports the resonance wavelength variations, in absolute value, calculated with respect to the configuration without volume in the tank versus the water volume in the tank. The linear fitting and the error bars, calculated as the maximum variation of the measured resonance wavelength (approx. 0.2 nm), are reported in the same figure.

From the results, it is possible to observe that the resonance wavelength is blue-shifted when the micro-volume around the unjacketed LDF-POF increases. In the analyzed range, the devised micro-volume sensor denotes a good linear response. The sensitivity and the resolution are estimated by using the linear function reported in Figure 10b, resulting in 0.337 nm/μL and 0.59 μL, respectively [36].

In the end, a comparative analysis is reported in Table 4, where the presented sensor system is compared with two volume liquid sensors, already presented in the literature, in terms of resolution. 

### 3.5. Two-Dimensional Micro Displacement Sensor

**Figure 11:**
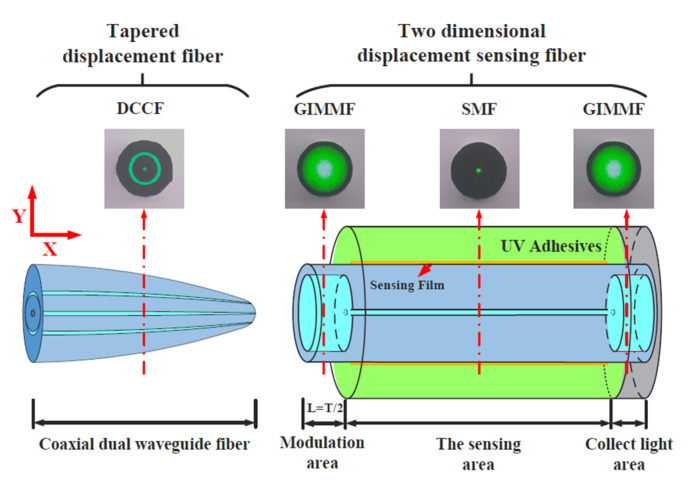
Structure of the two-dimensional micro displacement sensor system with a characterization of displacement and sensing probes [39].

The two-dimensional micro displacement sensor system consists of two parts connected in series: the first is a displacement probe realized by tapering a conical structure processed by a dual concentric-core fiber (DCCF), and the second part is the sensing probe realized by splicing two types of fiber. In particular, the sensing probe can be divided into three areas: the modulation area, realized by using a graded multimode fiber with a core diameter of 105 μm; the sensing area with a length of 2 cm, realized by using a single-mode fiber; and the light collecting area, realized by using the same type of graded multimodal fiber used for the modulation area [39]. The three parts are spliced in order to obtain the sensing probe. Finally, the sensing probe is covered by 50 nm of gold layer, followed by a UV-curable adhesive with a refractive index of 1.35. Figure 11 shows the sensor system with a characterization of the displacement probe and sensing probe.

**Figure 12:**
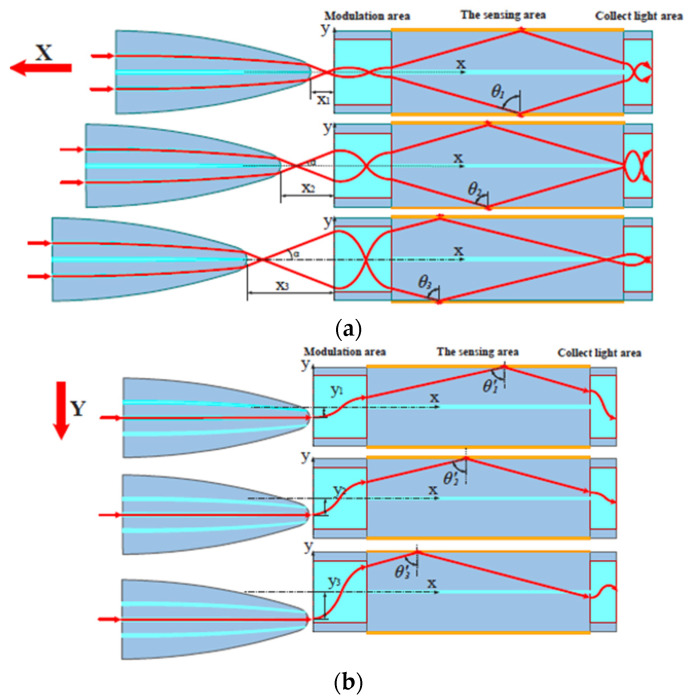
Outline of the sensing principle. Displacement variation along the (**a**) x-axes and (**b**) y-axes [39].

The sensing principle is the same as in the previously described sensors. The displacement probe moves along the x- and the y-axes; in this way, the propagating mode profile in the sensing probe varies and the modulation area emphasize the phenomenon. In other terms, the variation of the displacement produces a variation in the incident angles at the input of the sensing probe, thus inducing a shift in resonance wavelength. Figure 12 shows a schematization of the sensing principle of the described sensor system.

**Figure 13:**
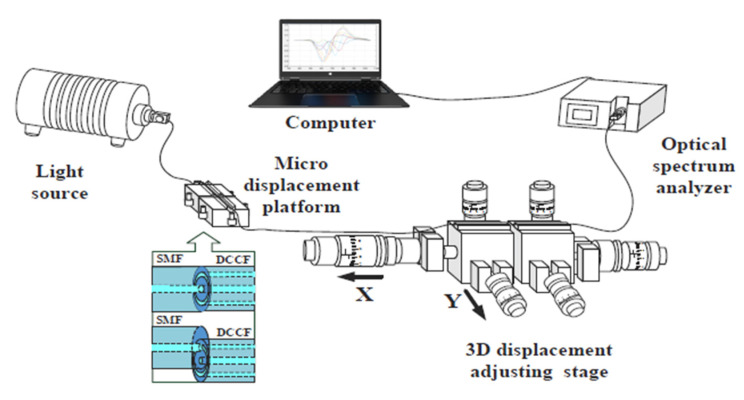
Experimental setup used to test the two-dimensional micro displacement sensor [39].

In order to test the two-dimensional micro displacement sensor system, an experimental setup is used based on a wide spectrum light as a source and an optical spectrum analyzer as a receiver. The sensor system is placed between the source and the receiver. In particular, the light is injected into a patch of the single mode fiber (SMF) that is coupled with the DCCF by using a fiber-coupling micro motion table. The tapered end of the displacement probe and the end of the modulation area of the sensing probe are fixed on the left and right, respectively, of the 3D displacement table (MP-225, Sutter) in order to change the displacement of the tapered end, along the x- and y-axes, with respect to the sensing probe. Finally, the transmitted light was gathered from the sensing probe by an optical spectrum analyzer [39]. Figure 13 reports a schematization of the experimental setup. More details about this two-dimensional micro displacement sensor are extensively reported in [39].

**Figure 14:**
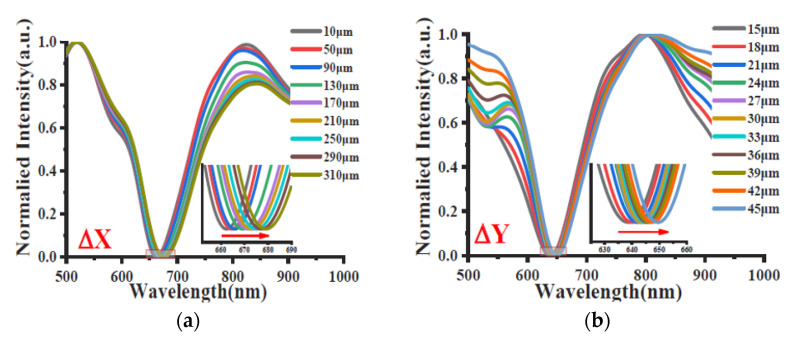
Normalized SPR spectra achieved by varying the displacement of the tapered probe along (**a**) the x-axis and (**b**) the y-axis [39].

The described sensor system is tested by moving the tapered displacement fiber first along the x-axis and then along the y-axis, while the refractive index on the SPR sensing probe is fixed to 1.35 by using the UV-curable adhesive. In particular, in the x-axis direction, the displacement varies in the range from 10 μm to 310 μm, with a step size of 40 μm; in the y-axis direction, the displacement varies from 15 μm to 45 μm, with increasing steps of 3 μm. Figure 14 shows the normalized SPR spectra achieved by varying the displacement as described. As is clear, in both cases, the resonance wavelengths increase with the displacement. 

**Figure 15:**
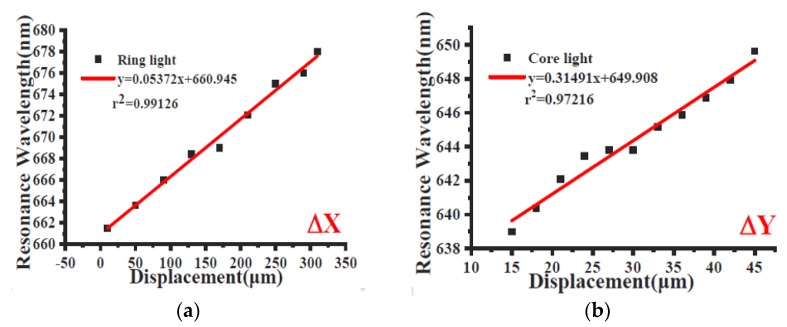
Experimental resonance wavelength versus the displacement along (**a**) the x-axis and (**b**) the y-axis [39].

Figure 15 shows the experimental resonance wavelength versus the displacement along the two axes (x and y). In more detail, the sensor response can be considered linear in the analyzed range along the x-axis (R^2^ = 0.99), while for the case of the y-axis, the linearity of the sensor is less pronounced (R^2^ = 0.97). Furthermore, by using the experimental results in Figure 15, the sensitivity equals 0.0537 nm/μm for the x-axis direction case and 0.315 nm/μm for the y-axis direction case [39]. 

In order to compare the presented sensor system performance with other experimental configurations already presented in the literature, a comparative analysis is carried out and reported in Table 5.

## 4. Bio/Chemical Sensors

The presented sensing approach is also exploited by realizing bio/chemical sensor systems. In this case, the part of the system sensitive to the analyte is a platform realized with a modified 1 mm POF. The latter is fixed onto a resin support and polished with two polishing papers (5 μm and 1 μm grits) in order to obtain the D-shaped area. After this, orthogonal micro-holes, with diameters of 600 μm, are drilled by using a computer numerical control (CNC) micro-milling machine. The microstructured POF is then functionalized by filling the micro-holes with a synthetic receptor for a specific analyte.

The operating principle is the same as for the previous sensor configurations: the functionalized microstructured POF is placed in series and used to launch the light into an SPR D-shaped platform, which presents a fixed refractive index solution as the encompassing medium. In this way, the analyte–receptor binding locally changes the effective refractive index of the core of the microstructured platform. This phenomenon produces a variation in the propagating mode of the input light in the plasmonic platform and, consequently, the resonance wavelength shifts.

The first application based on the described operating principle regards the detection of furfural (2-FAL) in water. The POF presents one micro-hole filled by a molecularly imprinted polymer (MIP), specific for the detection of 2-FAL [43]. The refractive index of the medium on the SPR platform is fixed at 1.332 (water), and different concentrations of 2-FAL are tested on the microstructured platform. In a similar way, this measurement approach is used to detect perfluorooctanoic acid (PFOA) in water. For this application, the microstructured platform presents three micro-holes filled by an MIP receptor, specific to PFOA [44]. 

**Figure 16:**
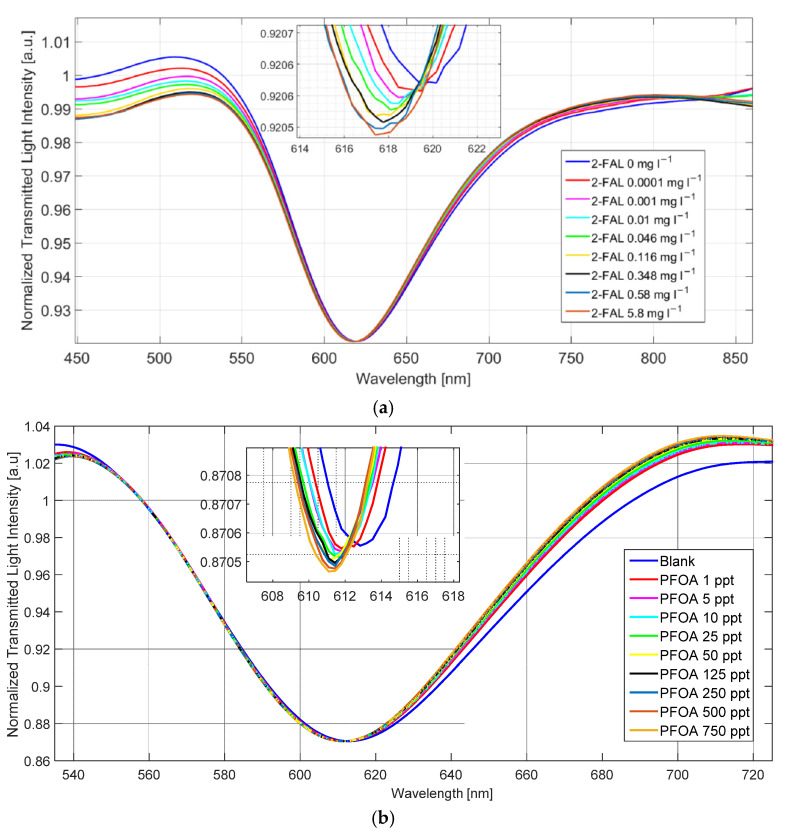
SPR spectra, obtained by a normalization of the reference spectrum, at different (**a**) 2-FAL [43] and (**b**) PFOA [44] concentrations in water. Reprinted with permission from Ref. [43]. Copyright 2022, Elsevier.

Figure 16 shows the SPR spectra achieved at different 2-FAL and PFOA concentrations, obtained by a normalization of the reference spectrum, acquired when air is the encompassing medium of the SPR platform and with water (blank solution) on the microstructured platform [43,44]. For both applications, the measurement protocol consists of 10 min of incubation and repeated washing with water in order to remove the nonspecific adsorption. From Figure 16, it is clear that, in both cases, the resonance wavelength decreases when the 2-FAL or PFOA concentration increases.

Table 6 reports, for both the mentioned applications, a comparative analysis in terms of limits of detection (LODs). In particular, the sensing configurations, which exploit the novel sensing approach, are compared with two similar configurations in which the same MIPs receptors are deposited directly on the gold surface of the same SPR-POF probe. In [43,44], the resin block used to fix the POFs, in order to realize the D-shaped POF sensing area with the micro-holes and to obtain the planar area to drop the under-test solution, is not influent for the transmission spectrum because the micro-holes present a depth less than the POF’s thickness in the D-shaped POF area.

From Table 6, it can be seen that the LOD improves by using the presented sensor systems [43,44] compared with the conventional functionalized SPR-POF platforms [45,46]. 

Furthermore, Table 7 and Table 8 report a comparative analysis of the different sensors already presented in the literature for the detection of 2-FAL and PFOA, respectively.

## 5. Conclusions

This review reported an innovative sensing method which overturned the traditional SPR technique by harnessing the properties of multimodal POF. The described sensor devices consisted of a sensitive patch coupled in sequence to a multimode waveguide-based plasmonic platform realized, for instance, by D-shaped POFs. It was reported how variations in the physical and chemical quantity of interest could be determined by exploiting the properties of the multimodal waveguides. Furthermore, it is important to stress that, by using this approach, the SPR platform was no longer required to interact directly with the measurand. Thus, the sensing approach reported in this review exceeded the limits of the conventional SPR sensors regarding the thickness of the sensitive layer over the SPR surface. Additionally, this approach allowed the realization of sensors with high performance, useful in various application fields, via low-cost and simple production processes.

In future work, this innovative sensing approach could be exploited not only to detect different analytes by changing the MIP deposited into the POF chemical chip but also for the measurements of several physical quantities by designing novel sensitive patches. Moreover, less expensive experimental setups based on LED and photodiodes could be used instead of a white light source and a spectrometer to reduce the entire sensor system cost.

## Figures and Tables

**Table 1:** A comparison of several magnetic field sensors based on different sensing methods.

Sensing Method	Sensitivity (pm/mT)	Resolution (mT)	Ref.
Based on altering the plasmonic conditions in multimode SPR-POF platform	6800	0.029	[23]
Based on a magnetic fluid as surrounding medium of a plasmonic optical-fiber-based platform	3000	-	[24]
Based on a magnetic fluid as surrounding medium of a plasmonic optical-fiber-based platform	10,000	0.5	[25]
Based on a magnetic fluid as surrounding medium of a plasmonic optical-fiber-based platform	870	-	[26]

**Table 2:** A comparison of several temperature sensors based on different sensing methods.

Sensing Method	Linear Range (°C)	Resolution (°C)	Ref.
Based on altering the plasmonic conditions in multimode SPR-POF platform	20–38	1.2	[27]
Based on a fiber optic displacement platform	42–90	2.4	[29]
Based on a Fabry–Perot interferometer	-	1	[30]
Based on plasmonic phenomenon in optical fibers	30–70	0.8	[31]

**Table 3:** A comparison of several force sensors based on different sensing methods.

Sensing Method	Resolution	Ref.
Based on altering the plasmonic conditions in multimode SPR-POF platform	~22 mN	[32]
Based on reflection measurements by optical fibers	~10 mN	[33]
Based on whispering-gallery-mode resonators	~10 µN	[34]
Based on intensity variation in a POF beam	~0.1 N	[35]

**Table 4:** A comparison of several volume sensors based on different sensing methods.

Sensing Approach	Resolution (μL)	Ref.
Based on altering the plasmonic conditions in multimode SPR-POF platform via unjacketed LDF patch	5.9	[36]
Based on bending losses phenomena in optical fiber	71	[37]
Based on bend sensor and linear extension spring	4	[38]

**Table 5:** Comparative analysis of different optical displacement sensors based on plasmonic techniques.

Sensor	Axis	Sensitivity	Detection Range	Ref.
Based on the coaxial double waveguide with a conical structure	X	0.0537 nm/µm	300 µm	[39]
	Y	0.315 nm/µm	30 µm	
Based on the graded multimode fiber with double V-grooves	X	0.148 nm/µm	240 µm	[40]
	Y	3.724 nm/µm	9 µm	
	Z	3.543 nm/µm	6 µm	
Based on Kretschmann configuration on graded-index multimode fiber (GIMMF)	Y	10.32 nm/μm	25 μm	[41]
Based on hetero-core structure of graded-index multimode fiber	Y	1.23 nm/μm	50 μm	[42]

**Table 6:** Comparative analysis of the proposed 2-FAL and PFOA sensor system and two other similar configurations based on the same receptor (MIP) and SPR-POF platform.

Sensor System	Detection	LOD	Ref.
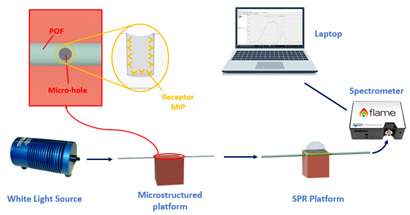	2-FAL in water	0.04 (μg/L)	[43]
	PFOA in water	8.1 × 10^−7^ (μg/L)	[44]
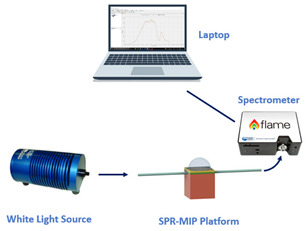	2-FAL in water	42.9 (g/L)	[45]
	PFOA in water	0.13 (μg/L)	[46]

**Table 7:** Comparative analysis of the proposed 2-FAL sensor system and others already presented in the literature based on the same receptor (MIP).

Sensor	Matrix Solution	Sensitivity at Low Conc. (nm/mg L^−1^)	Limit of Detection (μg/L)	Ref.
MIP-POF based on one micro-hole	Water	4084	0.04	[43]
MIP-POF based on three micro-holes	Water	6500	0.2	[43]
SPR-POF probe covered by MIPs	Water	31.0	42.9	[45]
MIP-POF based on three micro-holes	Milk		0.01	[47]

**Table 8:** Comparative analysis of the proposed PFAS sensor system and others already presented in the literature.

Sensing Method	Target Analyte	LOD (ppt)	Concentration Range (ppt)	Ref.
Fluorescence	PFOS	7.5 × 10^4^	0–10^9^	[48]
Electrochemical impedance spectroscopy	PFOS	1.7 × 10^3^	0–250	[49]
Ion-transfer stripping voltammetry	PFCAs, PFSAs	25 × 10^3^	0–500	[50]
Fabry–Perot Interferometry (FPI)	PFOA	5 × 10^6^	900–5000	[51]
SPR-POF-MIP	PFOA	130	0–4000	[46]
Intensity-based POF-MIP	PFOA	210	0–4000	[52]
SPR-POF chip and MIP-based chip	PFOA	0.86	0–750	[44]

## Data Availability

The data are available on reasonable request from the corresponding author.
